# Extraction spectrophotometry using a lithium-ion selective metallacrown: temperature effect on extraction reaction and application to determination of lithium in serum and seawater

**DOI:** 10.1007/s44211-024-00569-9

**Published:** 2024-04-23

**Authors:** Shoichi Katsuta, Kosuke Maeda

**Affiliations:** 1 Department of Chemistry, Graduate School of Science, Chiba University, 1-33 Yayoi-Cho, Inage, Chiba 263-8522 Japan; 2 Department of Chemistry, Graduate School of Science and Engineering, Chiba University, 1-33 Yayoi-Cho, Inage, Chiba 263-8522 Japan

**Keywords:** Lithium, Metallacrown, Extraction spectrophotometry, Serum, Seawater, Thermodynamics

## Abstract

A metallacrown-type ionophore, 2,3-pyridinediolate-bridged (3,5-dimethylanisole)ruthenium trinuclear complex, has a high extraction selectivity for Li^+^, but the extraction reaction is very slow. To solve this problem, the effect of temperature on the rapidity and equilibrium of the extraction of Li^+^ and Na^+^ as picrates from water to toluene with the metallacrown was investigated in this study. While the extraction of Li^+^ requires 6 h of shaking for equilibration at 25 °C, the distribution ratio becomes nearly constant after 4 h and 2 h of shaking at 37 °C and 50 °C, respectively. The extraction equilibrium constants (*K*_ex_) and associated thermodynamic parameters determined for Li^+^ and Na^+^ indicate that the extraction reactions are exothermic and enthalpy-driven: Δ*H*° = − 53 kJ/mol, Δ*S*° = − 0.03 kJ/(mol K) for Li^+^; Δ*H*° = − 28 kJ/mol, Δ*S*° = − 0.03 kJ/(mol K) for Na^+^. Although the extraction ability for Li^+^ and selectivity for Li^+^/Na^+^ decrease with increasing temperature, the values of *K*_ex_ and *K*_ex_(Li^+^)/*K*_ex_(Na^+^) are 1.0 × 10^7^ and 1.3 × 10^4^, respectively, even at 50 °C, indicating that both are sufficiently high. In the determination of Li^+^ by extraction spectrophotometry using this metallacrown, extraction at 50 °C for 2 h was employed to speed up the analysis. The method was applied to seawater and serum samples containing a large amount of coexisting ions such as Na^+^ and Mg^2+^, and trace amounts (10^−6^–10^−5^ mol/L order) of Li^+^ in microvolume samples (sub-mL order) could be successfully determined.

**Graphical abstract:**

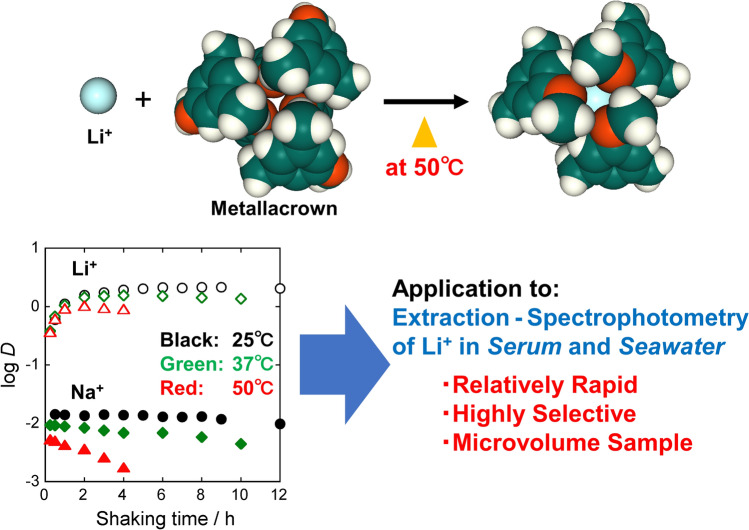

**Supplementary Information:**

The online version contains supplementary material available at 10.1007/s44211-024-00569-9.

## Introduction

In recent years, lithium-ion batteries have become indispensable for high-performance devices such as electric vehicles, renewable energy storage systems, and portable electric devices, and the demand for lithium as a raw material for these devices is rapidly increasing [1]. Lithium analysis is an essential step in understanding the resource and facilitating its effective utilization. Accurate and sensitive analytical methods are needed to characterize natural lithium deposits and establish efficient mining and production processes. Assessing the behavior of lithium in the environment and its impact on ecosystems is also critical to the sustainable management of lithium resources [2].

In addition, the potential of lithium in medical applications and new materials science is expanding. In the pharmaceutical field, lithium is known as a mood stabilizer and has been shown to be effective in the treatment of major depressive and bipolar disorders [3]. The effective blood concentration of lithium is 0.6–1.0 mmol/L. However, the effective blood concentration approaches the toxic range (generally > 1.5 mmol/L), requiring close monitoring of the blood lithium concentration [4].

Flame photometry, atomic absorption spectrometry, and ion electrode methods are generally used to measure lithium concentration in solution. In flame photometry and atomic absorption spectrometry, the matrix ions in the sample and the viscosity of the solution affect the measured value. Salt solutions containing high concentrations of matrix ions or serum samples with high viscosity often require dilution, resulting in reduced sensitivity. The ion electrode method uses the complexation reaction of lithium ions with a highly selective ionophore, such as dibenzyl-14-crown-4, for measurement [5, 6]. More recently, colorimetric methods utilizing the formation of F28-tetraphenylporphyrin complex with Li^+^ [7, 8] and LiKFe(IO_6_) complex [9] have also been reported. Although these chemical methods are useful as rapid and simple analytical methods, the selectivity between lithium and coexisting ions such as sodium and magnesium may be insufficient for some samples like brines.

**Fig. 1:**
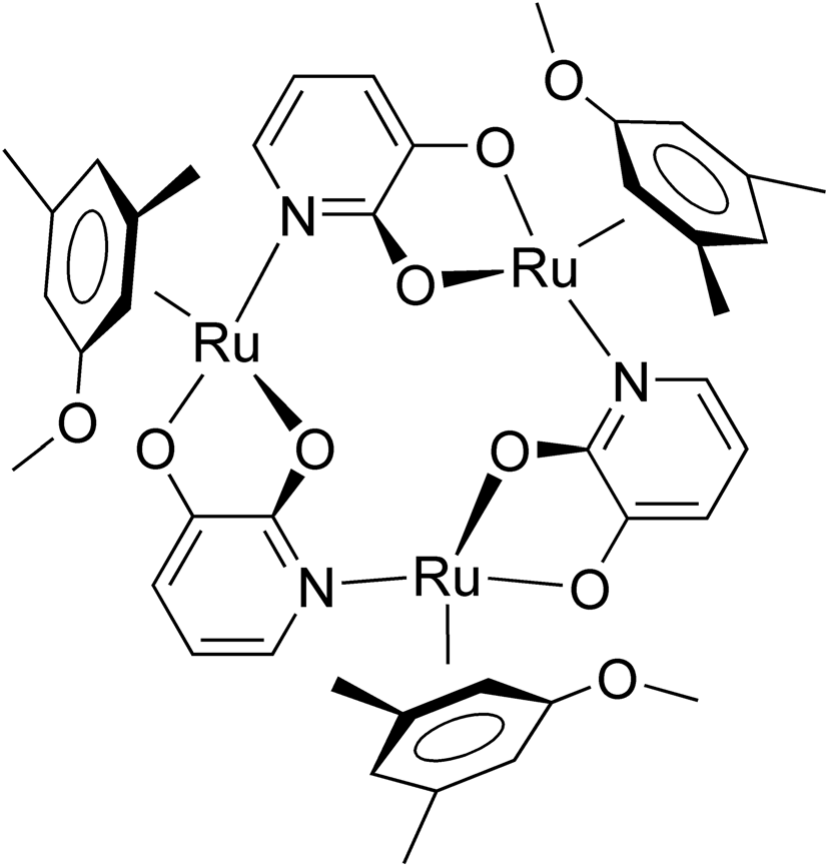
Structural formula of [{Ru(DMA)(pyO_2_)}_3_]

Previously, we synthesized a metallacrown compound possessing extremely high extraction selectivity toward Li^+^, [{Ru(DMA)(pyO_2_)}_3_] (Fig. 1), where DMA = 3,5-dimethylanisole and pyO_2_^2–^ = 2,3-pyridinediolate [10]. This compound behaves as an electrically neutral ligand (L) like a crown ether, forms a cationic complex with an alkali metal ion (M^+^), and extracts it into a nonpolar organic solvent as an ion pair with a hydrophobic monovalent anion (A^–^):

1 $${\text{M}}_{\text{w}}^+ + {\text{ L}}_{\text{o}} + {\text{ A}}_{\text{w}}^- \rightleftarrows {\text{MLA}}_{\text{o}}$$

where subscripts o and w denote organic phase and aqueous phase, respectively. The equilibrium constant for this extraction reaction (*K*_ex_) is defined as follows:

2 $$K_{{\text{ex}}} = \left[ {{\text{MLA}}} \right]_{\text{o}} /\left( {\left[ {{\text{M}}^+ } \right]_{\text{w}} \left[ {\text{L}} \right]_{\text{o}} \left[ {{\text{A}}^- } \right]_{\text{w}} } \right)$$

When A^–^ = picrate and organic solvent = dichloromethane, the *K*_ex_ values are 1.0 × 10^8^ and 1.4 × 10^3^ for Li^+^ and Na^+^, respectively, at 25 °C; the separation factor (*SF*_Li/Na_) defined as the ratio of *K*_ex_ for Li^+^ to that for Na^+^ is 7.1 × 10^4^. Furthermore, an extraction spectrophotometric determination method for Li^+^ was established using [{Ru(DMA)(pyO_2_)}_3_], and was applied to the determination of Li^+^ in seawater [11]. This method has excellent sensitivity and selectivity for Li^+^ determination, but its drawback is that extraction is very slow, requiring 12 h of shaking for extraction equilibration.

In this study, with the aim of speeding up this Li^+^ analysis method, the effects of temperature on the equilibration time of extraction and on the equilibrium constant and separation factor were investigated. Furthermore, the method was modified to shorten the analysis time and reduce the required sample volume, and its effectiveness was evaluated by applying it to the determination of Li^+^ in serum and seawater.

## Experimental

### Reagents and samples

The synthesis of [{Ru(DMA)(pyO_2_)}_3_], preparation of its toluene solution, and spectrophotometric determination of its concentration were carried out as described previously [10, 11]. Water was deionized with a Kurita Demi-Ace Model DX-15A demineralizer and further purified with a Millipore Simplicity UV System just before use. Toluene (GR grade, Kanto Chemical) was distilled and washed three times with pure water. An aqueous solution of lithium picrate was prepared by neutralization of lithium hydroxide monohydrate (GR grade, Wako Pure Chemical Industries) with picric acid (GR grade, Wako Pure Chemical Industries) in water, whereas an aqueous solution of sodium picrate was prepared using commercially available sodium picrate monohydrate (EP grade, Kanto Chemical). Lithium chloride (99.9%, Wako Pure Chemical Industries), sodium chloride (GR grade, Kanto Chemical), and potassium chloride (GR grade, Kanto Chemical) were dried at 250 °C for 4 h under reduced pressure. Bovine serum albumin lyophilized powder, purchased from Sigma-Aldrich, was used as received. All other reagents were of GR grade or higher and used without further purification.

An artificial serum was prepared as an aqueous solution containing 140 mol/L NaCl, 1.5 mmol/L KCl, 1.0 mmol/L MgCl_2_·6H_2_O, 2.5 mmol/L CaCl_2_·2H_2_O, 1.0 mmol/L NaH_2_PO_4_·2H_2_O, 1.0 mmol/L K_2_HPO_4_, 4.7 mmol/L d-glucose, 2.5 mmol/L urea, and 0.60 mmol/L bovine serum albumin. A standard serum JCTCM 131 (with lithium content of less than 0.1 mmol/L) was obtained from Reference Material Institute for Clinical Chemistry Standards. An artificial seawater was prepared according to JIS K 2510 [12]. These samples were used after adding appropriate amounts of LiCl. A natural seawater was collected on December 15, 2018 at Inage Beach, Japan (35°37′11″ N, 140°03′29″ E, WGS84), filtered through a 0.45 μm membrane filter of mixed cellulose esters, and stored at 4 °C until use.

### Instruments

A Hitachi Z-5000 polarized Zeeman atomic absorption spectrophotometer was used for flame photometry. The UV–visible spectrophotometers used were a Shimadzu UV-1800 for absorption spectrum measurements and a Beckman DU-640 with a 50 μL microcell having a light-path length of 10 mm for fixed wavelength absorbance measurements. Extraction (shaking) at different temperatures was performed with an As One MyBL-100CS block bath shaker in a rotating mode at 1350 rpm, and centrifugation was done with a Kubota 2010 tabletop centrifuge at 3000 rpm for 10-mL centrifuge tubes or an As One HSC-12000 high spin mini-centrifuge at 10,000 rpm for 1.5-mL microcentrifuge tubes.

### Stability test of the metallacrown

Equal volumes (2 mL) of water and a toluene solution of 1.9–2.0 mmol/L [{Ru(DMA)(pyO_2_)}_3_] were placed in a FEP 10-mL centrifuge tube and mechanically shaken for 0.5–12 h at 25, 37, and 50 (± 0.5) °C. After centrifugation for 5 min, a portion of the toluene phase was pipetted out, diluted 100-fold with toluene, and the UV–visible absorption spectrum was measured.

### Extraction of alkali metal picrates

Equal volumes (2 mL) of a toluene solution of 0.60–1.7 mmol/L [{Ru(DMA)(pyO_2_)}_3_] and an aqueous solution of 0.76 mmol/L lithium picrate or 12 mmol/L sodium picrate were placed in a FEP 10-mL centrifuge tube. The tube was mechanically shaken for 0.25–12 h at 25, 37, and 50 (± 0.5) °C. After centrifugation for 5 min, the concentration of Li or Na in the organic phase was determined as follows. One milliliter of the organic phase was transferred to a PFA beaker and evaporated to dryness under reduced pressure. One milliliter of concentrated nitric acid was added to the beaker to decompose the metallacrown, and the solution was again evaporated to dryness by heating. The residue was re-dissolved in 60 mmol/L hydrochloric acid and the alkali metal concentration was determined by flame photometry. The concentration of the alkali metal in the aqueous phase was determined by subtracting the concentration in the organic phase from the initial concentration in the aqueous phase. The distribution ratio (*D*) of the alkali metal was calculated as the ratio of the molar concentration in the organic phase to that in the aqueous one.

The procedure for the extraction spectrophotometric determination of Li^+^ in seawater and serum samples is described in the following section.

## Results and discussion

### Effect of temperature on the extraction reaction

**Fig. 2:**
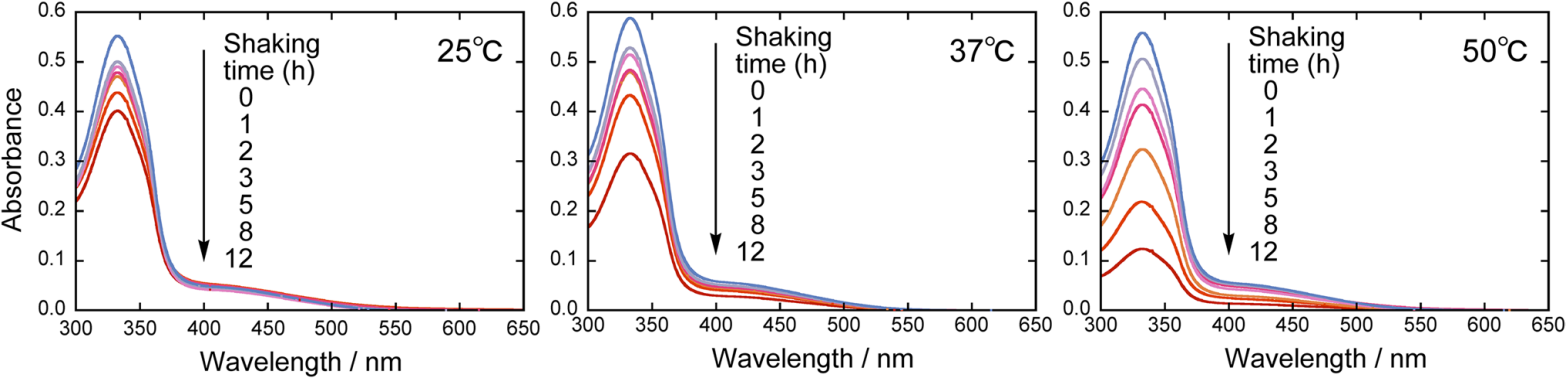
UV–Vis spectra of [{Ru(DMA)(pyO_2_)}_3_] in toluene before and after shaking with water at different temperatures (after 100-fold dilution with toluene). Initial concentration of [{Ru(DMA)(pyO_2_)}_3_] was 2.0 mmol/L at 37 °C and 1.9 mmol/L at 25 °C and 50 °C

**Fig. 3:**
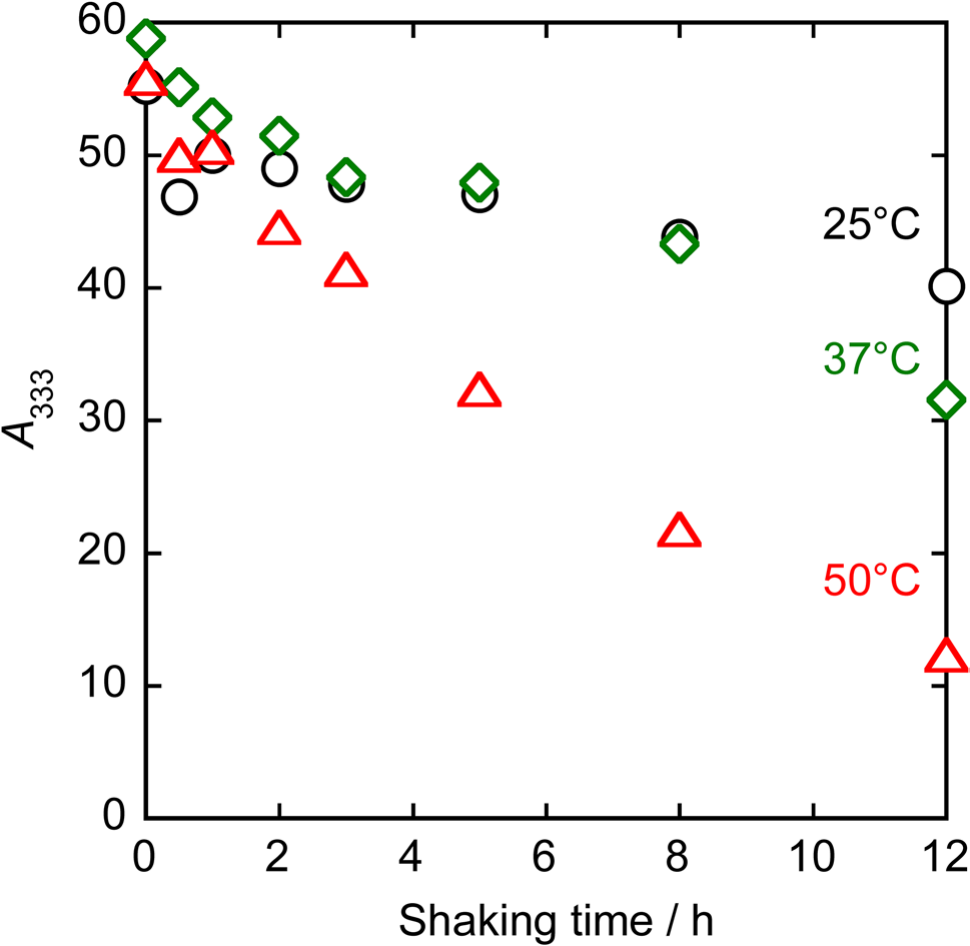
Shaking-time dependence of the absorbance at 333 nm (*A*_333_) of [{Ru(DMA)(pyO_2_)}_3_] in toluene after shaking with water at different temperatures (converted to pre-dilution values). Initial concentration of [{Ru(DMA)(pyO_2_)}_3_] was 2.0 mmol/L at 37 °C and 1.9 mmol/L at 25 °C and 50 °C

The stability of [{Ru(DMA)(pyO_2_)}_3_] in the toluene–water biphasic system was evaluated by measuring the UV–Vis spectra of a toluene solution of [{Ru(DMA)(pyO_2_)}_3_] before and after shaking with water. The absorption spectra of the toluene phase at 25 °C, after 100-fold dilution, are shown in Fig. 2. The absorbance decreases with increasing shaking time, but there is no significant change in the maximum absorption wavelength (333 nm) and the shape of the spectrum. The same holds true at 37 °C and 50 °C. Figure 3 shows the absorbance of the toluene phase at 333 nm (*A*_333_, converted to the value before dilution) as a function of shaking time at 25 °C, 37 °C, and 50 °C, respectively. At all temperatures, the *A*_333_ value decreases slightly from the pre-shaking absorbance (*A*_333,init_) immediately after shaking, and once it becomes approximately constant, indicating that the partitioning of [{Ru(DMA)(pyO_2_)}_3_] from the toluene phase to the aqueous phase has reached equilibrium. The distribution constant (*K*_D_) of [{Ru(DMA)(pyO_2_)}_3_], defined as the ratio of molar concentration in the toluene phase to that in the aqueous phase, was calculated as *K*_D_ = *A*_333_/(*A*_333,init_ – *A*_333_), with values of 7.1 ± 0.7 at 25 °C, 8 ± 2 at 37 °C, and about 9 at 50 °C, respectively. Here, the values of *A*_333_ used to calculate *K*_D_ were those at shaking time of 0.5–5 h at 25 °C and 37 °C, and 0.5–1 h at 50 °C. The tendency for the *K*_D_ value to increase with increasing temperature is consistent with the tendency observed in the distribution of 18-crown-6 between benzene and water [13]. The absorbance decreases again with shaking time when it exceeds about 5 h at 25 °C and 37 °C, and 1 h at 50 °C. These may be due to the decomposition of [{Ru(DMA)(pyO_2_)}_3_] in the aqueous phase; the percentage of [{Ru(DMA)(pyO_2_)}_3_] remaining in the toluene phase after 12 h of shaking is 73% at 25 °C, 54% at 37 °C, and 22% at 50 °C, showing that the decomposition is accelerated with increasing temperature.

**Fig. 4:**
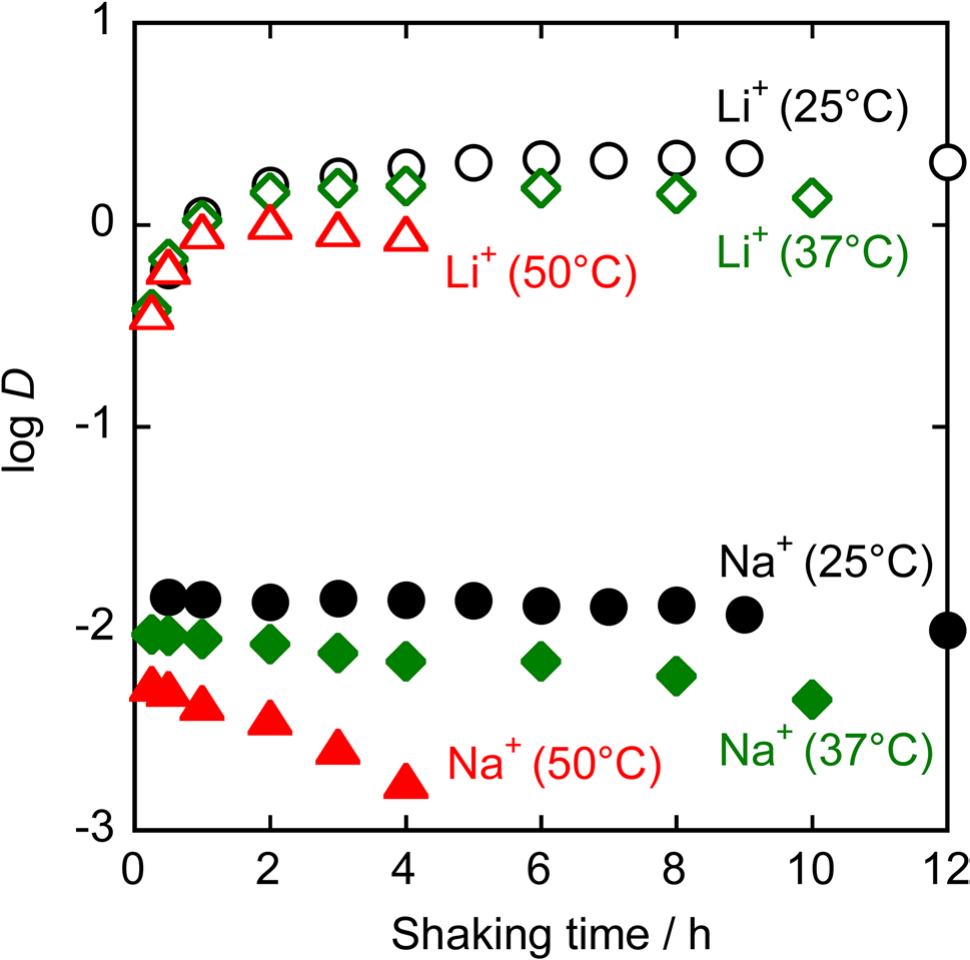
Shaking-time dependence of the distribution ratio of Li^+^ and Na^+^ at different temperatures in the extraction of alkali metal picrates with [{Ru(DMA)(pyO_2_)}_3_] in toluene. Initial concentrations of [{Ru(DMA)(pyO_2_)}_3_], lithium picrate, and sodium picrate were 0.75 mmol/L (in org. phase), 0.75 mmol/L (in aq. phase), and 12 mmol/L (in aq. phase), respectively

The dependence of Li^+^ and Na^+^ extraction on shaking time was evaluated by varying the shaking time while the initial concentrations of [{Ru(DMA)(pyO_2_)}_3_] (0.75 mmol/L) in the toluene phase and alkali metal picrate (0.75 mmol/L for Li^+^; 12 mmol/L for Na^+^) in the aqueous phase were kept constant. The log *D* values of alkali metal ions at 25 °C, 37 °C, and 50 °C are plotted against the shaking time in Fig. 4. The shaking time required for the log *D* of Li^+^ to become constant or maximum is 6 h at 25 °C, 3–4 h at 37 °C, and 2 h at 50 °C. The log *D* of Li^+^ at 50 °C clearly decreases with shaking time when the shaking time exceeds 2 h. This may be due to the faster decomposition of [{Ru(DMA)(pyO_2_)}_3_] than at other temperatures, as shown in Fig. 3. The log *D* of Na^+^ at 25 °C becomes nearly constant by shaking for 0.5 h or more. However, as the temperature increases to 37 °C and 50 °C, the decrease in log *D* of Na^+^ with shaking time becomes more pronounced. This is also attributable to the decomposition of [{Ru(DMA)(pyO_2_)}_3_].

Assuming that the extraction reaction of Eq. (1) proceeds and that alkali metal ion (M^+^) and picrate ion (A^–^) are almost completely dissociated in the aqueous phase and only exist as the ion pair MLA in the organic (toluene) phase, the distribution ratio *D* of M^+^ is expressed as follows:

3 $$D \approx \, \left[ {{\text{MLA}}} \right]_{\text{o}} /\left[ {{\text{M}}^+ } \right]_{\text{w}} = K_{{\text{ex}}} \left[ {\text{L}} \right]_{\text{o}} \left[ {{\text{A}}^- } \right]_{\text{w}}$$

Transforming this equation yields Eq. (4):

4 $${\text{log }}\left( {D/\left[ {{\text{A}}^- } \right]_{\text{w}} } \right) = {\text{log }}\left[ {\text{L}} \right]_{\text{o}} + {\text{log }}K_{{\text{ex}}}$$

[A^–^]_w_ and [L]_o_ can be calculated based on the mass balance as [A^–^]_w_ = [A]_t_ − [MLA]_o_ and [L]_o_ = ([L]_t_ − [MLA]_o_)/(1 + 1/*K*_D_), respectively, where the subscript t denotes the total concentration.

**Fig. 5:**
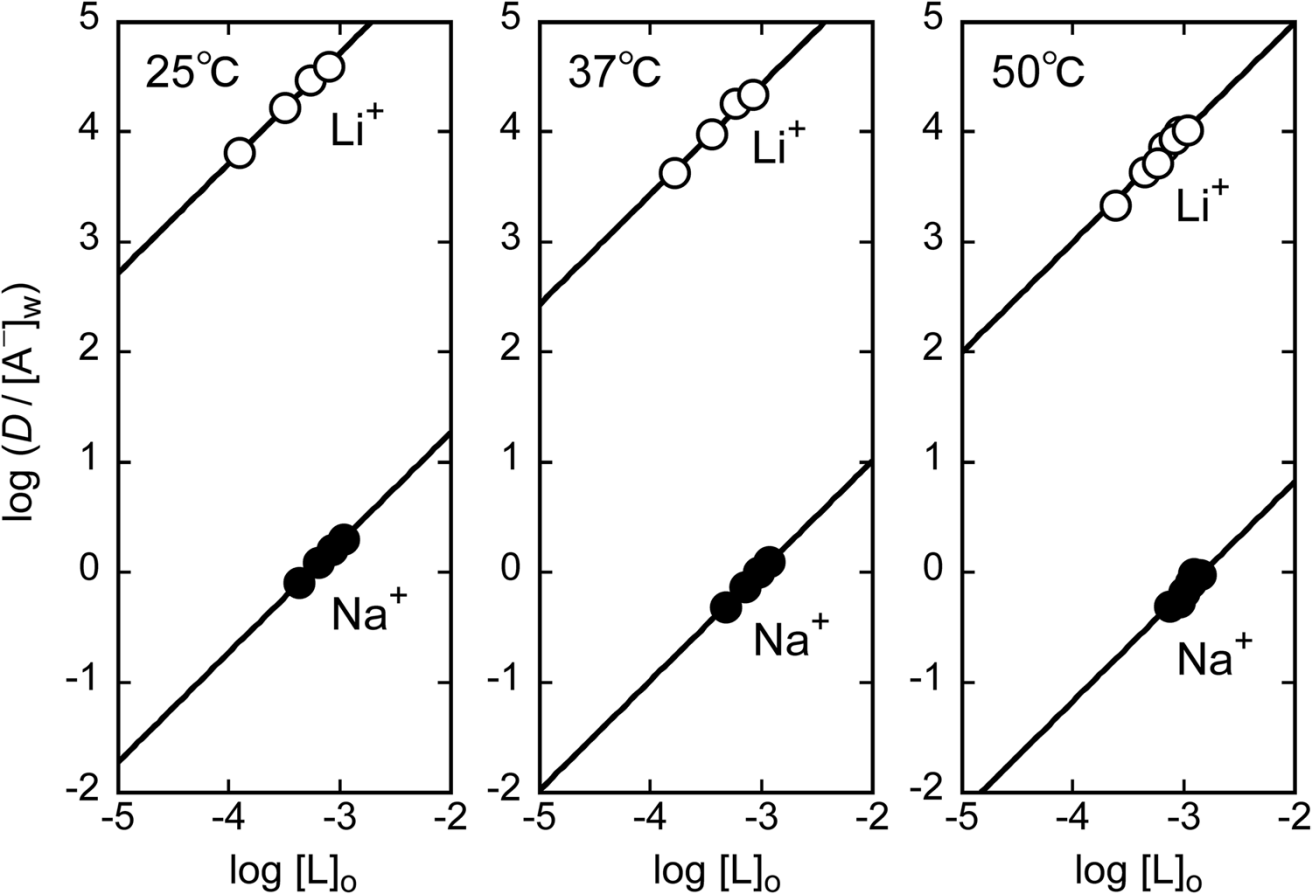
Plots of log (*D*/[A^–^]_w_) vs. log [L]_o_ at different temperatures in the extraction of alkali metal picrates with a toluene solution of [{Ru(DMA)(pyO_2_)}_3_]

Figure 5 shows log (*D*/[A^–^]_w_) vs. log [L]_o_ plots using extraction data obtained with shaking times of 6 h at 25 °C, 4 h at 37 °C, at 2 h at 50 °C, where the influence of decomposition of L is small or negligible. The slope of the straight line is 0.99 for both Li^+^ and Na^+^ at 25 °C, 1.06 for Li^+^ and 1.04 for Na^+^ at 37 °C, and 1.11 for Li^+^ and 1.22 for Na^+^ at 50 °C, indicating that Eq. (4) is valid. Substituting the values of *D*, [L]_o_, and [A^–^]_w_ into Eq. (3), the *K*_ex_ values at each temperature were calculated. The results are summarized in Table 1, together with the separation factor *SF*_Li/Na_. The *K*_ex_ values at 37 °C and 50 °C may be somewhat imprecise, i.e., apparent constants, due to the decomposition of the metallacrown.

**Table 1:** Extraction equilibrium constants at different temperatures

Temperature/ °C	Log *K*_ex_		*SF*_Li/Na_
	Li^+^	Na^+^	
25	7.719 (0.009)	3.278 (0.003)	2.77 × 10^4^
37	7.44 (0.02)	3.022 (0.004)	2.6 × 10^4^
50	7.00 (0.02)	2.90 (0.04)	1.3 × 10^4^

Values in parenthesis are standard errors

The value of *K*_ex_ decreases with increasing temperature, showing that this reaction is exothermic. Since this tendency is greater for Li^+^ than for Na^+^, the Li^+^/Na^+^ selectivity also decreases with increasing temperature. However, the *K*_ex_ and *SF*_Li/Na_ values at 50 °C, 1.0 × 10^7^ and 1.3 × 10^4^, respectively, indicate that the Li^+^ extractability and Li^+^/Na^+^ selectivity are still sufficiently high.

The thermodynamic parameters of the extraction reaction were evaluated from the temperature dependence of *K*_ex_. The − *R* ln *K*_ex_ vs. 1/*T* plot [Fig. S1 (supplementary information)] shows a good linear relationship for each alkali metal ion as expected from the van’t Hoff equation (− *R* ln *K*_ex_ = Δ*H*°/*T* − Δ*S*°). The thermodynamic parameters calculated are as follows: Δ*H*° = − 53 ± 7 kJ/mol, Δ*S*° = − 0.03 ± 0.02 kJ/(mol K) for Li^+^; Δ*H*° = − 28 ± 6 kJ/mol, Δ*S*° = − 0.03 ± 0.02 kJ/(mol K) for Na^+^. At 25–50 °C (298–323 K), the extraction reaction is enthalpy-driven because the absolute value of Δ*H*° is larger than that of *T*Δ*S*°. The Δ*S*° values of both metal ions are equal, indicating that the difference in Δ*G*° between Li^+^ and Na^+^ is dominated by the difference in Δ*H*°. This suggests that the difference in binding energy of Li^+^ and Na^+^ with the metallacrown is the main cause of the extraction selectivity. When compared to the thermodynamic parameters of a typical crown ether extraction system, the 18-crown-6–potassium picrate–benzene system (log *K*_ex_ = 5.39 at 25 °C, Δ*H*° = − 77 kJ/mol, Δ*S*° = − 0.14 kJ/(mol K) [13]), the [{Ru(DMA)(pyO_2_)}_3_]–lithium picrate–toluene system has a larger *K*_ex_ value due to the smaller entropy change in the extraction reaction.

### Brush-up of the extraction spectrophotometric Li^+^ determination method and its application to serum and seawater samples

In view of the temperature effect on the extraction reaction, the extraction spectrophotometric method for determination of Li^+^ proposed earlier [11] was modified to speed up the process. At the same time, a scale-down of the method was also considered to reduce the sample amount required. The modified method is as follows [the schematic illustration of the method is shown in Fig. S2 (supplementary information)]:

- [Step 1] *Preparation of aqueous phase for extraction*: Add an aqueous solution of sodium picrate to the sample solution and dilute with water as needed to bring the concentration of picrate concentration to 12 mmol/L.
- [Step 2] *Extraction of Li*^+^: Place 0.5 mL of the aqueous solution prepared in Step 1 and 0.5 mL of a toluene solution containing 2.0 mmol/L of [{Ru(DMA)(pyO_2_)}_3_] in a 1.5-mL microcentrifuge tube made of PFA. Shake the tube for 2 h at 50 °C by a block bath shaker. Allow the tube to stand at room temperature (25 °C) for 10 min, then centrifuge for 5 min.
- [Step 3] *Phase cleaning*: Transfer 0.4 mL of the toluene phase to another PFA 1.5-mL microcentrifuge tube containing the same volume of deionized water. Shake the tube for 10 min at 25 °C, then centrifuge it for 5 min.
- [Step 4] *Coloration and absorbance measurement*: Transfer 0.3 mL of the toluene phase to another PFA 1.5-mL microcentrifuge tube containing the same volume of an aqueous solution of 0.10 mmol/L potassium tetrabromophenolphthalein ethyl ester (K[TBPE]), shake the tube for 10 min at 25 °C, centrifuge it for 5 min, and measure the absorbance of the toluene phase at 571 nm.

The extraction operations in Steps 2–4 are performed for selective and quantitative extraction of Li^+^ to the toluene phase, removal of the co-extracted metal ions such as Na^+^ and Mg^2+^ from the toluene phase, and exchange of the counter ions (A^–^) in the ion pair (MLA) from picrate to TBPE^–^ (i.e., color development), respectively [11]. In this study, the shaking times in Steps 2–4 were re-examined using artificial seawater as a sample and found to be sufficient for their respective purposes. The extraction conditions of Step 2 yielded more than 95% extraction of Li^+^ for artificial seawater samples with pH adjusted from 4.0 to 8.6 with hydrochloric acid, potassium hydrogen phthalate, potassium dihydrogen phosphate, boric acid, and sodium hydroxide [results shown in Fig. S3 together with the details of the experimental procedure (supplementary information)].

**Fig. 6:**
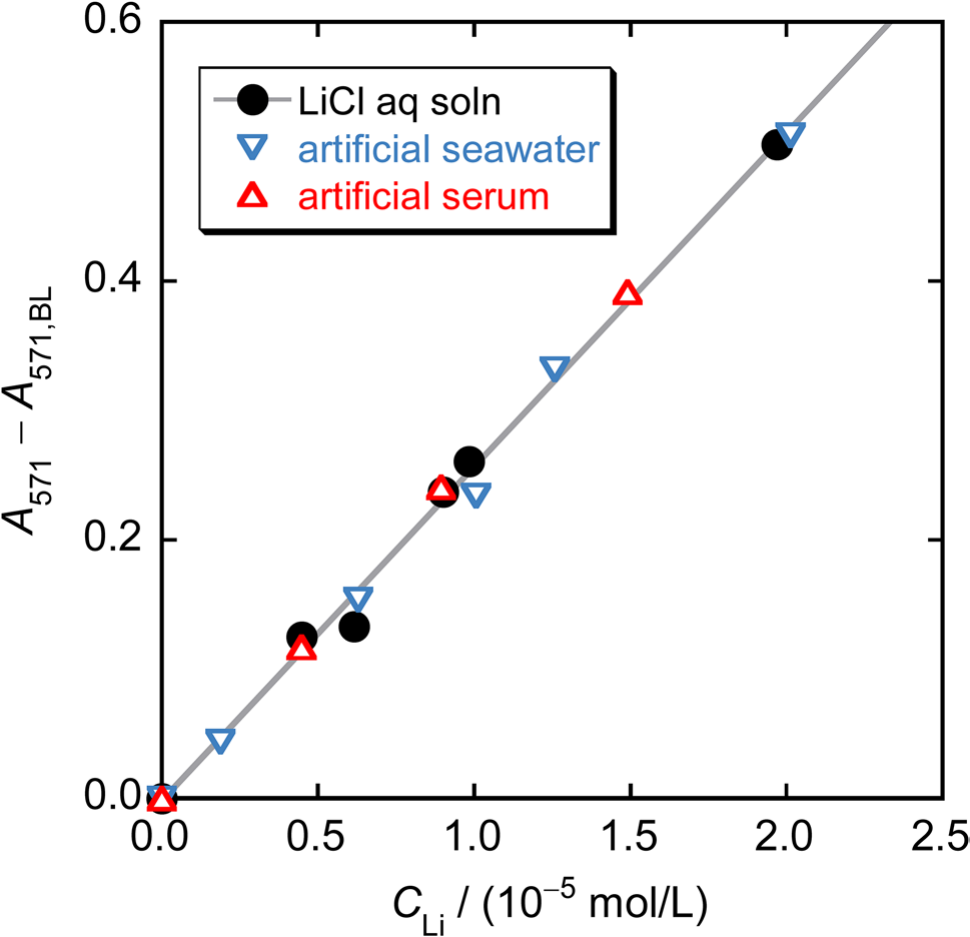
Relationship between the absorbance at 571 nm of the toluene phase in Step 4 and the concentration of Li^+^ in the aqueous phase prepared in Step 1 when using aqueous LiCl solution, artificial seawater, and artificial serum as sample solutions. Artificial seawater and artificial serum were diluted 2.5- and 125-fold, respectively, in Step 1

The method was applied to aqueous lithium chloride solutions of different concentrations, and the absorbance of the toluene phase at 571 nm (*A*_571_) minus that of the blank (*A*_571,BL_) plotted against the concentration of Li^+^ in the aqueous phase prepared in Step 1 (*C*_Li_) is shown in Fig. 6. A good linear relationship is observed with a slope of 25.8 L/mmol. The limits of detection (LOD, *k* = 3) and quantification (LOQ, *k* = 10) were evaluated from triple measurements of *A*_571,BL_ (*ca.* 0.6): LOD = 1.6 × 10^−3^ mmol/L and LOQ = 5.2 × 10^−3^ mmol/L. Similar experiments were performed with artificial seawater and artificial serum with different concentrations of LiCl. Herein, the seawater and serum samples were diluted 2.5- and 125-fold, respectively, in Step 1; thus, the minimum volumes of seawater and serum used for a single analysis are 0.2 mL and 4 μL, respectively. The extraction temperature of 50 °C in Step 2 is lower than the denaturation temperature of serum proteins [14]. The *A*_571_ – *A*_571,BL_ vs. *C*_Li_ plots obtained are also shown in Fig. 6, indicating that the coexisting components in seawater and serum do not affect the relationship between *A*_571_ − *A*_571,BL_ and *C*_Li_ at all. As mentioned earlier [11], the blank absorbance (*A*_571,BL_) depends on the concentrations of [{Ru(DMA)(pyO_2_)}_3_] and K[TBPE] used in Steps 2 and 4, respectively, while it is almost independent of the sample matrix. Therefore, the following equation can be used as a calibration line:

5 $$C_{{\text{Li}}} /\left( {{\text{mmol}}/{\text{L}}} \right) \, = \, \left( {A_{{571}} -A_{{571},{\text{BL}}} } \right)/{25}.{8}$$

where *A*_571,BL_ is practically the absorbance at 571 nm when using water as the sample solution in Step 1 that measured in the same run with the measurements of *A*_571_.

The analytical values of Li^+^ when this method was applied to a natural seawater and a standard serum added with 0.82 mmol/L LiCl are shown in Table 2. Here, as in the calibration line preparation, the seawater and serum samples were diluted 2.5- and 125-fold in Step 1, respectively. The analytical values for the same samples by flame photometry using the standard addition method are also shown in the Table 2. The analytical values of Li^+^ in the natural seawater by the two methods are in good agreement with each other. On the other hand, the analytical values of Li^+^ in the standard serum differ slightly between the two methods; however, the values by this method and the frame photometry are 107% and 96% of the added Li^+^, respectively, which are both acceptable as the results of spike and recovery test.

**Table 2:** Analytical results of Li^+^ in real samples

Sample	Li^+^ concentration^a^/ mmol/L	
	This method	Flame photometry^b^
Natural seawater	0.027 (0.002)	0.0279 (0.0004)
Standard serum with LiCl added ^c^	0.88 (0.03)	0.79 (0.04)

^a^Values in parenthesis are standard errors from three independent measurements

^b^Obtained by the standard addition technique

^c^Concentration of LiCl added: 0.82 mmol/L

The LOQ value of 5.2 × 10^−3^ mmol/L for this method shown above corresponds to a serum Li^+^ concentration of 0.65 mmol/L before 125-fold dilution, which is well below the boundary between medical and toxic concentrations of Li^+^ (1.0–1.5 mmol/L) described in “Introduction”. This lower limit of concentration can be further lowered by reducing the dilution factor of the sample.

## Conclusions

A metallacrown [{Ru(DMA)(pyO_2_)}_3_] is a neutral ligand with extremely high extraction ability and selectivity for Li^+^, but the very slow extraction has been a bottleneck in its analytical use. This study found that increasing the extraction temperature reduces the extraction equilibration time from 6 h (25 °C) to 2 h (50 °C). The temperature dependence of the extraction equilibrium constant suggests that the extraction reaction is exothermic and that the binding energies of the metallacrown to Li^+^ and Na^+^ govern the extraction ability and selectivity for these metal ions. The extractability of Li^+^ and the selectivity for Li^+^/Na^+^ decrease with increasing temperature, but even at 50 °C they are sufficiently high. Therefore, by setting the extraction temperature to 50 °C, we succeeded in speeding up the analysis of the extraction spectrophotometric determination of Li^+^ using this metallacrown previously developed by the authors. This method is applicable to the determination of trace Li^+^ in high-matrix samples such as seawater and serum. The amount of sample required for a single analysis depends on the Li^+^ content, but is generally on the order of only 1 to 100 μL.

### Supplementary Information

Below is the link to the electronic supplementary material.

Supplementary file1 (DOCX 174 KB)

## Data Availability

Data are available based on the request.
