# Supplementary material for: Extraction spectrophotometry using a lithium-ion selective metallacrown: temperature effect on extraction reaction and application to determination of lithium in serum and seawater
Source: Anal Sci. 2024 Apr 23;40(7):1373–9. doi: 10.1007/s44211-024-00569-9 (PMC11208259; doi:10.1007/s44211-024-00569-9)
Supplement: Supplementary file 1 — Supplementary file1 (DOCX 174 KB) [file 44211_2024_569_MOESM1_ESM.docx]

Supplementary Information

**Extraction spectrophotometry using a lithium-ion selective metallacrown: temperature effect on extraction reaction and application to determination of lithium in serum and seawater**

Shoichi Katsuta^1^, Kosuke Maeda^2^

* Shoichi Katsuta

katsuta@faculty.chiba-u.jp

^1^ Department of Chemistry, Graduate School of Science, Chiba University, 1-33 Yayoi-cho, Inage, Chiba 263-8522, Japan

2 Department of Chemistry, Graduate School of Science and Engineering, Chiba University, 1-33 Yayoi-cho, Inage, Chiba 263-8522, Japan





**Fig. S1** Van’t Hoff plot for the extraction reaction of lithium and sodium picrates with a toluene solution of [{Ru(DMA)(pyO_2_)}_3_]

**

**

**Fig. S2** Schematic illustration of the extraction-spectrophotometric method for determination of Li^+^ in aqueous samples proposed in this study





**Fig. S3** Extraction percentage of Li^+^ as a function of aqueous pH in the extraction of lithium picrate from artificial seawater with a toluene solution of [{Ru(DMA)(pyO_2_)}_3_] at 50°C

**Experimental for Fig. S3:**

The aqueous buffer solutions prepared were a mixture of potassium hydrogen phthalate solution and hydrochloric acid, a mixture of potassium hydrogen phthalate solution and NaOH solution, a mixture of potassium dihydrogen phosphate solution and NaOH solution, and a mixture of boric acid solution and NaOH solution. Here, the concentration of the components of each solution before mixing was about 10 mmol/L. Aqueous solutions for extraction at pH 3.5 – 8.6 were prepared by mixing 3 mL of any of the above buffers, 1 mL of LiCl solution (0.12 mmol/L), 2 mL of sodium picrate solution (60 mmol/L), and 4 mL of artificial seawater.

0.5 mL of the above aqueous solution and 0.5 mL of a toluene solution of [{Ru(DMA)(pyO_2_)}_3_] (2.0 mmol/L) were placed in a PFA 1.5-mL microcentrifuge tube and mechanically shaken for 2 h at 50 (± 0.5)°C. The tube was then allowed to stand at 25°C for 10 min and centrifuged for 5 min. A 0.4 mL portion of the toluene phase was transferred to a PFA beaker and evaporated to dryness under reduced pressure. One mL of concentrated nitric acid was added to decompose the metallacrown, and the solution was evaporated to dryness by heating. The residue was dissolved in 60 mmol/L hydrochloric acid and the concentration of Li^+^ was measured by ICP emission spectrometry (PerkinElmer Avio 500) to determine the extraction percentage.
